# Supplementary figures and images for: Effect of field strength on RF power deposition near conductive leads: A simulation study of SAR in DBS lead models during MRI at 1.5 T—10.5 T
Source: PLoS One. 2023 Jan 26;18(1):e0280655. doi: 10.1371/journal.pone.0280655 (PMC9879463; doi:10.1371/journal.pone.0280655)

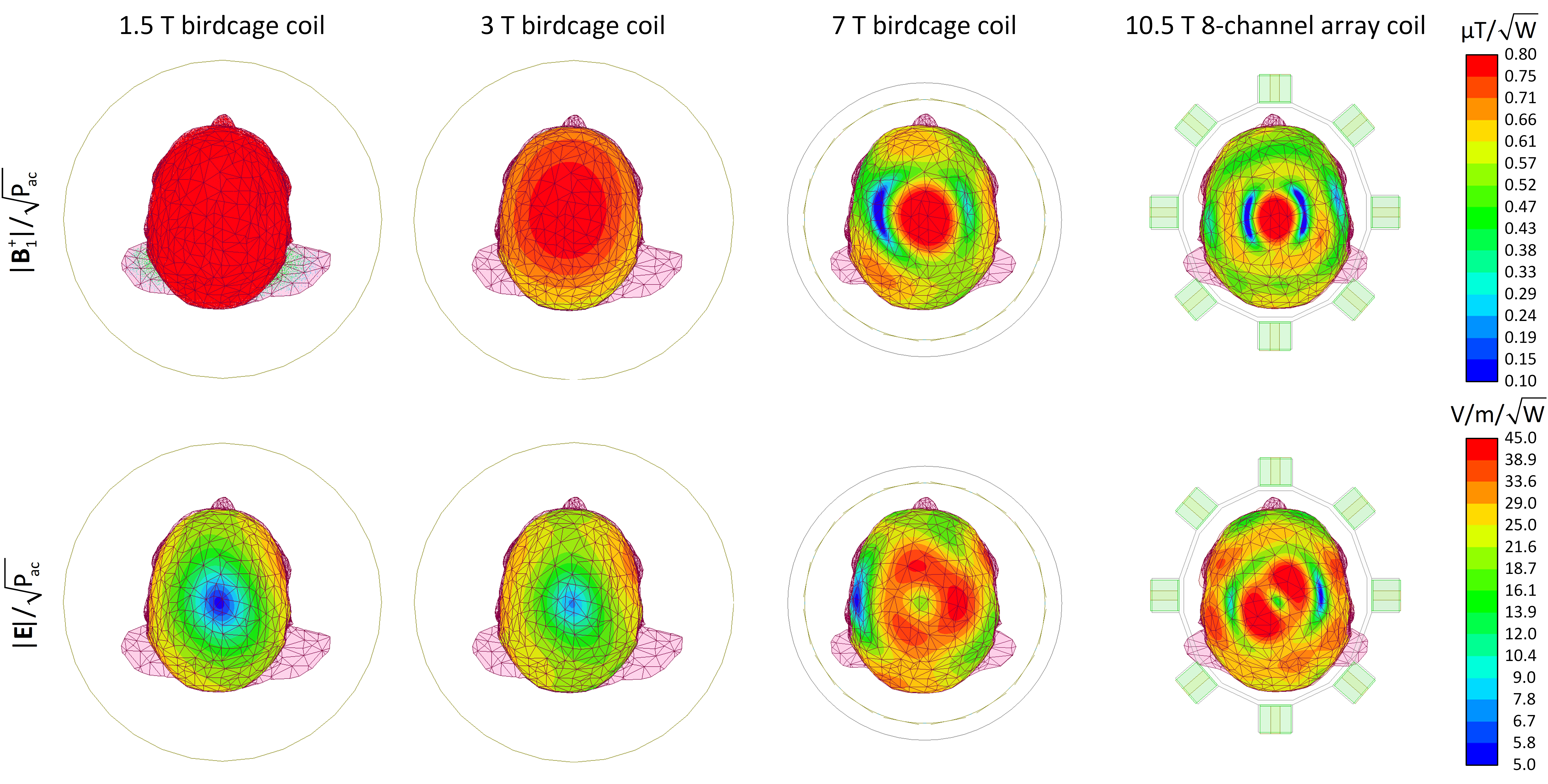

Supplement: S1 Fig — (PNG) [file pone.0280655.s001.png]
