# Supplementary material for: Effect of field strength on RF power deposition near conductive leads: A simulation study of SAR in DBS lead models during MRI at 1.5 T—10.5 T
Source: PLoS One. 2023 Jan 26;18(1):e0280655. doi: 10.1371/journal.pone.0280655 (PMC9879463; doi:10.1371/journal.pone.0280655)
Supplement: S1 Table — (DOCX) [file pone.0280655.s002.docx]

| Coil type | Field strength |  | When **B**_1_^+^ = 2 µT | | When GHSAR = 3 W/kg | |
| --- | --- | --- | --- | --- | --- | --- |
|  |  |  | 1g-SAR_max_ (W/kg) | GHSAR (W/kg) | 1g-SAR_max_ (W/kg) | **B**_1_^+^ (µT) |
| Birdcage coil | 1.5 T | Mean | 12.59 | 0.37 | 103.78 | 5.74 |
|  |  | SD | 12.14 | 0.04 | 98.51 | 0.33 |
| Birdcage coil | 3 T | Mean | 16.67 | 1.58 | 32.20 | 2.77 |
|  |  | SD | 15.35 | 0.16 | 29.67 | 0.15 |
| Birdcage coil | 7 T | Mean | 29.05 | 4.70 | 18.64 | 1.60 |
|  |  | SD | 23.86 | 0.22 | 15.23 | 0.04 |
| 8-channel array coil | 10.5 T | Mean | 51.04 | 5.27 | 29.13 | 1.51 |
|  |  | SD | 31.11 | 0.09 | 17.69 | 0.01 |
